# Effect and mechanism of microRNAs on various diabetic wound local cells

**DOI:** 10.1111/1753-0407.13474

**Published:** 2023-09-07

**Authors:** Hongjie Li, Shengyu Jing, Hongbo Xu

**Affiliations:** ^1^ Department of Vascular Surgery The Third Xiangya Hospital of Central South University Changsha China; ^2^ Central South University Xiangya School of Medicine Changsha China

**Keywords:** diabetic wound, local wound cell, microRNA, wound healing, microRNA, 伤口愈合, 糖尿病伤口, 局部伤口细胞

## Abstract

The difficulty of wound healing in diabetes mellitus has long been regarded as a thorny problem in the medical field. One of the important reasons is the abnormal function of wound‐related cells. A large number of recent studies have shown that microRNA (miR), a noncoding RNA that exists in eukaryotic cells, is closely linked to the functions of various cells in diabetic wound, and ultimately affects the healing of wound. This paper establishes for the first time the connection between miR and wound healing from the cellular perspective and summarizes the effects of various miRs on one or more kinds of wound cells, including their targets and related mechanisms. The abnormal expression of miRs in the wound has certain value for the early diagnosis of diabetic wounds. Moreover, it seems that correcting miRs that are abnormal expressed in the wound or artificially adding miRs that can promote wound healing has an essential therapeutic value.

## Graphical abstract

**Highlights**

- For the first time, we have examined the impact of microRNA on diabetic wounds at a cellular level.
- We have analyzed how a particular microRNA influences different types of cells, uncovering its function and mechanism.
- This study furthers the discovery of the clinical potential of diverse microRNAs by evaluating their distinct effects and mechanisms on multiple cell types.

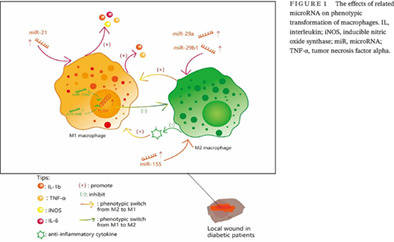

## 摘要

糖尿病创面愈合困难一直被认为是医学界的棘手问题。创面相关细胞功能异常是其重要原因之一。最近大量研究表明，microRNA (miR)是一种存在于真核细胞中的非编码RNA，与糖尿病创面中各种细胞的功能密切相关，并最终影响创面的愈合。本文从细胞角度建立miR与创面愈合之间的联系，并总结了各种miR对一种或多种创面细胞的作用，包括其作用靶点和相关机制。创面中miR的异常表达对糖尿病创面的早期诊断具有一定价值。此外，纠正创面中异常表达的miR或人为添加可促进创面愈合的miR似乎具有重要的治疗价值。

## 1 INTRODUCTION

As quality of life improves, the prevalence of diabetes is on the rise. According to the US Centers for Disease Control and Prevention, in 2018, a total of 34.2 million Americans were diagnosed with diabetes, representing 10.5% of the US population.^1^ Additionally, diabetics have a lifetime risk of 25% for developing foot ulcers, which is a major health concern and often results in hospitalization or amputation.^2^ Data analysis revealed that in 2014, the total treatment costs of diabetic foot ulcers (DFUs) in the United States amounted to $4499.9 million, signifying a serious social and economic burden.^3^ Compared with normal wounds, diabetic wounds are more difficult to heal. Because of the unknown mechanisms that impede wound healing, there is currently a lack of effective treatments. Thus, it is extremely important to unveil the mechanism behind refractory diabetic wounds and develop effective therapeutic drugs based on relevant knowledge.

The various stages of the normal wound healing process involve several local wound cells, such as macrophages, fibroblasts, keratinocytes, and vascular endothelial cells (ECs). After the platelets have successfully achieved hemostasis, macrophages play a crucial role during the initial inflammatory period of wound healing. During the beginning of the inflammation stage, macrophages mainly secrete relevant inflammatory factors, and in the later stage, they undergo a phenotype switch from M1 to M2, which allows them to carry out anti‐inflammatory and repair functions, thus the wound transitions from the inflammatory stage to the proliferative repair stage.^4^ In addition to the proliferative and repair functions of M2 macrophages, angiogenesis in the wound depends on the proliferation, migration, and branching of ECs. Subsequently, fibroblasts cooperate with these new blood vessels to form granulation tissue through their own proliferation and migration.^5^ At the same time, keratinocytes proliferate and migrate to the wound,^6^ promoting the differentiation of fibroblasts into myofibroblasts.^7^ The final remodeling stage is characterized by cross‐linking of extracellular matrix (ECM) secreted by myofibroblasts.^8^ However, in diabetic wounds, the level of inflammatory factors is significantly increased. Meanwhile, the high sugar environment also leads to various cell senescence, and then the cell function is impaired to varying degrees.^9^ After macrophage M1 had difficulty in transforming into M2 phenotype, the wound remained in the inflammatory stage for a long time, and it was difficult to transition to the repair stage. After the function of fibroblasts and keratinocytes is damaged, the proliferative repair stage and remodeling stage of the wound are obstructed, thus greatly prolonging the healing time of the wound. Angiogenesis, led by vascular ECs, plays an important role in all stages of wound healing, and its damaged function will inevitably prolong wound healing time. Therefore, how to restore the normal function of wound cells is particularly important for promoting diabetic wound healing.

Recent studies have revealed that microRNAs (miRs) ubiquitously expressed in eukaryotic cells play a significant role in multiple cell types in the wound. Adding numerous miRs and their analogues to the wound can effectively enhance wound healing. Moreover, many researchers have also noticed the abnormally expressed miRs in the local wound, circulation, and various cells of diabetic patients. Among them, miRs that play an important role include *miR‐20b‐5p*, *miR‐155*, *miR‐210*, *miR‐21‐3p*, *miR‐106b‐5p*, and *miR‐221‐3p*. By acting on specific intracellular targets, miRs interfere with numerous signaling pathways, thereby impeding the normal function of cells and ultimately leading to related dysfunctions of multiple cells in the wound. This paper presents an overview of the effects of different miRs from wounds, circulation, cells, or artificial addition on multiple cells in wounds, mainly from the perspective of local cells in diabetic wounds. It is hoped that the development of appropriate regulatory drugs for the abnormal expression of miRs in the local wound, circulation, or various cells of diabetic patients according to different modes of action, or the artificial introduction of drugs containing miRNA or miRNA mimics to the wound, ultimately result in improved diabetic wound healing.

## 2 OVERVIEW OF MICRORNA

MicroRNA is a small noncoding RNA that plays an important role in post‐transcriptional gene regulation.^10^ There are many forms of miR. Pri‐miRNA, the most primitive miR, is processed once and becomes pre‐miR, namely miR precursor, and pre‐miR is digested by Dicer enzyme to become mature miR.^11^ A single miR can target hundreds of mRNAs, regulate the post‐transcriptional silencing of target genes, and then affect a variety of cellular functions through epigenetic ways,^12^ including cell growth, differentiation, development, apoptosis, and other cellular activities.^11^ As for the relationship between miRNA and diabetes, several studies have shown that miR is closely related to wound healing, liver glucose and lipid metabolism,^13^ retinopathy,^14^ diabetic nephropathy,^15^ coronary artery and heart function,^16^ and so forth. In diabetic wounds, many studies have found that a variety of miRs are abnormal expressed locally and in circulation, which affect cell proliferation, migration, and apoptosis by abnormal regulation of many downstream targets, including toll‐like receptor 4 (TLR4) gene in macrophages, calcineurin 1 (RCAN1) in fibroblasts, fibrillin‐1 (FBN1) in keratinocytes, phosphatase and tensin homolog (PTEN) in vascular ECs, and so on. After the intervention of abnormal miRNA expression, various types of cells have shown significant functional improvement. Therefore, various abnormally expressed miRNAs in wound both has the value of early diagnosis of abnormal wound healing and the potential to be the targets for promoting wound healing.

## 3 MIRS AND MACROPHAGES

On the local wound surface of diabetes mellitus, the imbalance between M1 and M2 phenotype of macrophages has been reported in many studies, which is manifested as increased M1 and decreased M2.^17^, ^18^ This situation leads to the fact that the proinflammatory effect dominated by M1 is greater than the proregeneration effect dominated by M2 in diabetic wounds, so that wounds are in the inflammatory stage for a long time and cannot transition to the granulation repair stage.

### 3.1 MiRs altered in local diabetic wounds

The expression of related miRs is abnormal in local diabetic wounds, leading to phenotypic transformation disorders of macrophages. *MiR‐21*, closely related to M1, was shown to be upregulated in the early stage of the wound (inflammation stage), which would increase the expression of M1‐related inflammatory factors interleukin (IL)‐1β, tumor necrosis factor alpha (TNF‐α), inducible nitric oxide synthase, and IL‐6.^17^

A recent control experiment found that *miR‐29a* and *miR‐29b1* were increased in diabetic skin wounds compared with the normal control group, and with the upregulation of M1 polarization, the expressions of related inflammatory factors IL‐1b and TNF‐α also appeared.^18^ Furthermore, *miR‐29ab1* knockout mice have shown superior wound healing and attenuated imflammation,^18^ which suggested that *miR‐29ab1* has the potential to be a drug target and improve wound healing.

Several studies have shown that *miR‐155* expression is increased in diabetic wounds,^19^, ^20^ which promotes the conversion of macrophages to M1 phenotype by inhibiting negative regulators of inflammation.^21^ All of the aforementioned miRs are upregulated in diabetic wounds. Detection of these upregulated miRs is helpful for early diagnosis of refractory diabetic wounds, and targeting these miRs or related inflammatory factors can effectively improve wound inflammation and accelerate wound healing.^18^, ^19^, ^20^, ^21^

### 3.2 Altered miRs in circulating macrophages

Most wound macrophages are recruited and differentiated from the circulating monocytes/macrophages, and abnormal miR expression of bone marrow derived monocytes/macrophages can lead to phenotypic transformation disorder of wound macrophages. A report by Xuefeng Peng and colleagues found that with the expression of *miR‐146a*, negatively regulating TLR4 is reduced and TLR4 increased in circulating monocytes/macrophages of diabetic patients, thus inhibiting phenotype switch to M2.^22^ Similarly, another study showed that in type 2 diabetic mice, the expression of *miR‐448* in circulating monocytes/macrophages was significantly decreased, resulting in the weakening of the inhibitory effect of *miR‐448* on TLR4. Subsequent upregulation of TLR4 promoted transformation of macrophages to M1 phenotype.^23^ Taken together, downregulation of miRs negatively regulatingTLR4 in circulating monocytes/macrophages contributes to M1 retention of wound macrophages. Adding *miR‐146a* and *miR‐448* mimics has the ability to inhibit the expression of TLR4 and promote the phenotypic transformation of M1 macrophages.

**FIGURE 1:**
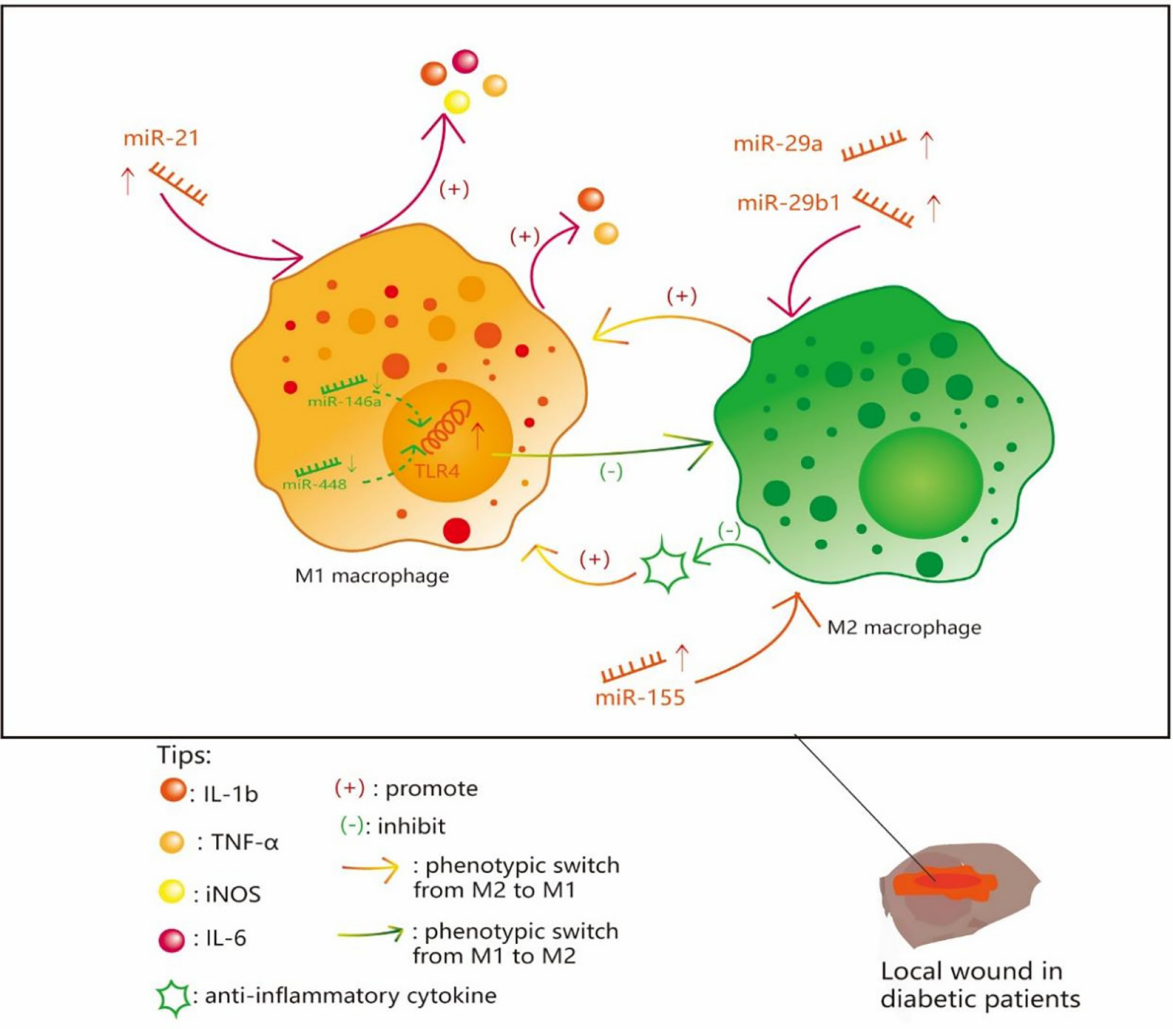
The effects of related microRNA on phenotypic transformation of macrophages. IL, interleukin; iNOS, inducible nitric oxide synthase; miR, microRNA; TNF‐α, tumor necrosis factor alpha.

Here we summarize the effects of related miRs on phenotypic transformation of macrophages (Figure 1).

### 3.3 Exogenous intervention based on miR to regulate macrophage function

Some artificially added exogenous miR mimics can also regulate macrophage phenotype switching. Bahram Saleh and colleagues first demonstrated that *miR‐223* mimic could act on macrophages to inhibit the expression of proinflammatory genes and promote the expression of anti‐inflammatory genes through in vitro experiments.^24^ Further in vivo experiments showed that hydrogel loaded with *miR‐223* mimic promoted the formation of blood vessels and skin in chronic wounds, including diabetic wounds, and the main mechanism was to promote the transformation of macrophages to M2 phenotype.^24^ Therefore, *miR‐223* mimic combined with appropriate vector has the potential to effectively reduce diabetic wound inflammation and promote the transition of wound to repair period. Modern studies have found that after photobiomodulation and allogeneic diabetic adipose tissue‐derived stem cells are applied to diabetic local wounds, they can increase the content of *miR‐146a* on wound surface. The production of inflammatory cells (neutrophils, macrophages) and IL‐1 was also reduced, which ultimately improved the inflammatory phase of wound healing in type I diabetic rats and promoted the proliferation of wound repair.^25^ These findings indicate that therapeutic regimen based on the macrophage‐related miRs has great potential to improve the local inflammation of the wound and promote wound healing.

## 4 MIRS AND FIBROBLASTS

Some research found miRs have certain effects on fibroblasts in diabetic wounds, such as *miR‐92a‐3p* ^26^ and *miR‐181a‐5p*.^27^ Therefore, local fibroblast dysfunction is another important reason for impaired diabetic wound healing, which is mainly manifested as the inhibition of proliferation and migration, the increase of apoptosis, and the reduction of extracellular matrix and cytokines secreted, leading to the damage of granulation repair process and prolonging healing time.

### 4.1 Abnormal miRs in local wounds

The function of fibroblasts could be modulated by the abnormal expressed miRs in the wound. *MiR‐103*, significantly increased in the wound margin tissues of diabetic patients, could diminish RCAN) expression in human skin fibroblasts (HSFs), thereby inhibiting HSF proliferation and migration while promoting apoptosis.^28^ Downregulation of *miR‐103* mitigated hyperglycemia‐induced HSF injury by promoting RCAN1 expression, so it can promote wound repair by inhibiting the expression of *miR‐103*.^28^ Another study found that *miR‐27‐3p* was overexpressed in the wounds of diabetic mice and patients. The decreased level of its downstream target neuro‐oncological ventral antigen 1 in fibroblasts was shown to cause less proliferation and extracellular matrix production but more apoptosis.^29^ Inhibition of *miR‐27‐3p* can restore fibroblast activity, implying the therapeutical potential. In the diabetic mice models conducted by Yaohong Wu and colleagues, *miR‐21‐3p* was significantly decreased on the fourth and seventh day after wound establishment compared with normal control. Upregulation of sprout homolog 1(SPRY1), the downstream target of *miR‐21‐3p*, can suppress the proliferation of fibroblasts, enhance autophagy, and reduce collagen synthesis.^30^ After treatment with *miR‐21‐3p* agonist, fibroblast function was enhanced by reducing SPRY1, and a rapid wound healing process was achieved in mice, which has the potential to treat DFUs.^30^ Additionally, it was shown that high glucose environment in diabetic wounds could inhibit hypoxia‐induced *miR‐210* expression and impair downstream hypoxia‐inducible factor 1 (HIF‐1) signaling, which induces decreased glycolysis, increased oxygen consumption, and reactive oxygen species (ROS) production of HSFs.^31^ Interestingly, *miR‐210* reconstruction improve wound healing via normalizing glycolysis and ROS level.

### 4.2 Circulating exosomal miRs

In diabetic patients, circulating exosomes carrying over‐expressed *miR‐20b‐5p* can be transferred into HSFs and induce HSFs dysfunction by suppressing vascular endothelial growth factor A (VEGFA).^32^ Thus *miR‐20b‐5p* could be a biomarker and promising target for early prevention and diagnosis of refractory diabetic wounds.

### 4.3 LncRNA/miR pathways

MiRs are also involved in the formation of long noncoding RNA (lncRNA)–mediated pathways regulating fibroblasts, among which lncRNA cancer susceptibility candidate 2 (CASC2)/miR‐155/HIF‐1α is well studied.^19^ Compared with control patients, *miR‐155* was significantly higher in DFUs, with the lower level of lncRNA CASC2 and HIF‐1α. This dysregulated pathway dampened the migration, proliferation, and collagen production of fibroblasts.^19^ Another study reported that in the margin of DFUs *miR‐29b* was downregulated; its upstream lncRNA H19 and downstream FBN1 were upregulated; whereas these changes had positive effects on fibroblast functions, including increased proliferation and migration and decreased apoptosis.^33^ It is considered that these changes are attributed to compensatory effects of the body to promote the healing of diabetic chronic wounds.

### 4.4 MiRs associated with fibroblast reprogramming

Recently, a new method toward refractory diabetic wounds arises as reprogramming fibroblasts with abnormal functions from diabetic wounds and then applying them to the wound site to improve healing. Through reprogramming, intracellular expression of *miR‐29c‐3p* and *miR‐196a‐5p* was downregulated.^34^ Conversely, the expression of *miR‐197‐3p* and *miR‐331‐3p* was upregulated after reprogramming, along with the decreased expression of their targets caveolin‐1 and cyclin‐dependent kinase inhibitor 3 respectively. Together, these changes could promote fibroblast migration and ECM remodeling and potentially modulate fibrinolysis.^34^ These results suggest that the reprogramming technology for intracellular miRs may be applied to the clinical treatment of intractable diabetic wounds in the future.

Table 1 summarizes the specific effects and mechanisms of various miRs mentioned previously on fibroblasts.

**TABLE 1:** The specific effects and mechanisms of various miRs on fibroblasts.

MicroRNA	Source	Change of volume	Target or downstream substances	Change of downstream substances	Proliferation	Apoptosis	Migration	Affect extracellular matrix	Wound healing time	Supplement
*miR‐103* ^28^	Wound	↑	RCAN1	↓	−	/	−	/	Prolong	
*miR‐21‐3p* ^35^	Wound	↓	SPRY1	↑	−	+	/	−	Prolong	
*miR‐27‐3p* ^29^	Wound	↑	NOVA1	↓	−	+	/	−	Prolong	
*miR‐210* ^31^	Wound	↓	HIF‐1	↓	−	/	−	/	Prolong	
*miR‐155* ^19^	Wound	↑	HIF‐1α	↓	−	+	−	−	Prolong	Inhibition Upstream substance lncRNA CASC2 expression
*miR‐29b* ^33^	Wound	↓	FBN1	↑	+	−	+	/	Shorten	The compensatory effect of the wound
*miR‐20b‐5p* ^32^	Circulating exosomes	↑	VEGFA	↓	−	+	/	−	Prolong	
*miR‐29c‐3p* ^34^	Fibroblast	/	/	/	/	/	−	−	Prolong	Indirect reflect
*miR‐196a‐5p* ^34^	Fibroblast	/	/	/	/	/	−	−	Prolong	Indirect reflect
*miR‐197‐3p* ^34^	Reprogramming fibroblast	/	CAV1	↓	/	/	−	/	Prolong	Indirect reflect
*miR‐331‐3p* ^34^	Reprogramming fibroblast	/	CDKN3	↓	/	/	−	/	Prolong	Indirect reflect

*Note*: Tips: “↑”: increase, “↓”: decrease, “–”: inhibited, “+”: enhanced, “/”: have no effect or have not been reported in relevant literature.

Abbreviations: CASC2, cancer susceptibility candidate 2; CAV1, caveolin‐1; CDKN3, cyclin‐dependent kinase inhibitor 3; FBN1, fibrillin‐1; HIF‐1, hypoxia‐inducible factor 1; NOVA1, neuro‐oncological ventral antigen; RCAN1, calcineurin 1; SPRY1, sprout homolog 1;VEGFA, vascular endothelial growth factor A.

## 5 MIRS AND KERATINOCYTES

Keratinocytes are principally involved in the tissue formation stage of wound healing. The functions of these cells, such as migration and proliferation, are restricted in the wounds of diabetic patients, thereby impeding the process of wound epithelization and collagen remodeling and eventually hindering wound healing.

### 5.1 Abnormal miRs expression in the wound and plasma

Several studies showed that *miR‐203* has a great impact on the function of keratinocytes in wounds. Overexpressed *miR‐203* was identified in both plasma and wound margin tissues of diabetic patients, which disrupted proliferation and migration of keratinocytes via inhibiting p63.^36^ Negative pressure wound therapy can reduce the expression of *miR‐203*, promote DFU healing, and is expected to become an important means for the treatment of diabetic wounds.^36^ Additionally, another mice study proved that, through IL‐8/AKT pathway, upregulated *miR‐203* prolong the epithelial‐to‐mesenchymal transition process.^37^ These results show that correcting the overexpression of *miR‐203* could normalize the function of keratinocytes, thereby improving wound healing.

The downregulated miRs targeting keratinocytes include *miR‐31‐5p*, *miR‐12*, *miR‐335*, *miR‐204‐3p*, and *miR‐146a*. The decrease of *miR‐31‐5p* in full‐thickness wounds of diabetic mice relieved the downstream target FBN1 level and blocked keratinocytes migration.^38^, ^39^ The suppressed levels of *miR‐129* and *miR‐335* increased the expression of their common direct target Sp1 and Sp1/matrix metalloproteinase‐9 axis could inhibit migration of keratinocytes.^40^ Overexpression of *miR‐129* or *miR‐335* can improve the function of keratinocytes, suggesting that the combination of *miR‐129* and *miR‐335* has potential therapeutic value.^40^ Xiaotong Zhao and colleagues found that *miR‐204‐3p* was significantly decreased in both the circulating blood and the wound of DFU patients, whereas expression of zinc finger protein Kruppel like factor 6, negatively targeted by *miR‐204‐3p*, was increased, resulting in decreased proliferation and migration ability of keratinocytes and increased autophagy.^41^ Multifactorial logistic regression analysis showed that decreased expression of *miR‐204‐3p* in peripheral blood was an independent risk factor for DFU，which provides a potential therapeutic target.^41^ In another in vitro DFU model, the expression of *miR‐146a* was decreased in human keratinocytes, with increased expression of its target A‐kinase anchoring protein 12 (AKAP12), thereby inhibiting the proliferation and migration of keratinocytes by blocking HIF‐1α/Wnt/β‐catenin axis.^42^

In addition to affecting the classical repair function of keratinocytes, some miRs abnormally expressed in keratinocytes can also lead to the production of inflammatory responses. The levels of *miR‐17* to *92* clusters such as *miR‐19a/b* and *miR‐20a*, targeting SHCBP1 and SEMA7A respectively, were reduced in keratinocytes at the edge of human chronic wounds. Ultimately, the TLR3‐mediated nuclear factor kappa B (NF‐kB) pathway was activated to initiate the production of inflammatory chemokines and cytokines.^43^ In contrast, wound closure could be accelerated in vivo by conditional overexpression of the *miR‐19b* alone or *miR‐17* ~ *92* cluster in mice keratinocytes.^43^

### 5.2 Exogenous miR mimics

Qijun Lv and his colleague applied human adipose‐derived stem cell‐derived exosomes (hASC‐exos) loaded with *miR‐21‐5p* mimics to diabetic wounds, and found that *miR‐21‐5p* mimic promote the proliferation and migration of keratinocytes by activating the Wnt/β‐catenin pathway.^44^ Therefore, the combination of partial miR mimics with stem cell‐derived exosomes is expected to be an effective means to improve diabetic wound healing.

Table 2 summarizes the specific effects and mechanisms of various miRs mentioned on keratinocytes.

**TABLE 2:** The specific effects and mechanisms of various miRs on keratinocytes.

MicroRNA	Source	Change of volume	Target or downstream substances	Change of downstream substances	Proliferation	Apoptosis	Migration	Wound healing time	Supplement
*miR‐31‐5p* ^38^, ^39^	Wound	↓	FBN1	↑	/	/	−	Prolong	
*miR‐129* ^40^	Wound	↓	Sp1	↑	/	/	−	Prolong	By Sp1/MMP‐9 axis
*miR‐335* ^40^	Wound	↓	Sp1	↑	/	/	−	Prolong	By Sp1/MMP‐9 axis
*miR‐204‐3p* ^41^	Wound, circula‐tion	↓	KLF6	↑	−	+	−	Prolong	
*miR‐203* ^36^, ^37^	Wound, circula‐tion	↑	p63, IL‐8/AKT	↓	−	/	−	Prolong	
*miR‐146a* ^42^	HaCaT	↓	AKAP12	↑	−	/	−	Prolong	Inhibit HIF‐1α/
									Wnt/β‐catenin axis
*miR‐21‐5p* ^44^	hASC‐exos	/	Wnt/β‐ catenin	↑	+	/	+	Shorten	Function of miR‐21‐5p mimics
*miR‐19a/b* ^43^	Keratinocyte in wound margin	↓	SHCBP1	Through the activation of TLR3‐mediated NF‐kB pathway, promote the production of inflammatory chemokines and cytokines.					
*miR‐20a* ^43^	Keratinocyte in wound margin	↓	SEMA7A						

*Note*: Tips: “↑”: increase, “↓”: decrease, “–”: inhibited, “+”: enhanced, “/”: have no effect or have not been reported in relevant literature.

Abbreviations: AKAP12, A‐kinase anchoring protein; FBN1, fibrillin‐1; hASC‐exos, human adipose‐derived stem cell‐derived exosomes; HIF‐1, hypoxia‐inducible factor 1; IL, interleukin; miR, microRNA; KLF6, Kruppel like factor 6; MMP‐9, matrix metalloproteinase; NF‐κB, nuclear factor kappa B; TLR3, toll‐like receptor 3.

## 6 EFFECTS AND MECHANISMS ON ECS

Vascular epithelial cells are mainly involved in the granulation formation stage and tissue shaping stage of wound repair. In diabetic wounds, there are also various abnormal expressions of miRs that affect the related functions of vascular ECs, ultimately suppressing wound angiogenesis and delaying wound healing.

### 6.1 Abnormally expressed miRs in the wound

A recent study revealed that the expression of *miR‐152‐3p* was significantly increased at 4 and 9 days after wound formation in diabetic mice, which inhibited the expression of its downstream PTEN, thereby enhancing vascular EC apoptosis and reducing proliferation and tube formation The inhibition of *miR‐152‐3p* may effectively accelerate wound healing, thereby providing a potential target for DFU therapy.^45^

Another study showed that the expression of *miR‐199a‐5p* was significantly increased in the wound tissue of diabetic patients, the skin of diabetic rats, and cells induced by high glucose. By directly targeting VEGFA and Rho‐associated kinase 1 (ROCK1), it leads to decreased proliferation and migration of ECs.^46^ In diabetic rats, inhibition of *miR‐199a‐5p* significantly increased the expression of VEGFA and ROCK1, thus promoting wound healing. Therefore, *miR‐199a‐5p* and its targets have a significant therapeutic effect on diabetic wounds.^46^

Compared with normal mice, *miR‐31‐5p* was significantly reduced in the full‐thickness wound of diabetic mice, and its target HIF1AN was inadequate, which led to the deficiency of EC proliferation, migration, and tube formation.^38^ By using milk exosomes loaded with *miR‐31‐5p* mimics, the research team effectively improved the function of ECs in vitro and also promoted wound blood vessel formation and wound healing in this way.^38^

In the extracellular vesicles isolated from local diabetic foot wound effusion, many abnormal levels of miRs can also appear. Specifically, increased levels of *miR‐195‐5p* and *miR‐205‐5p* were discovered, and these two miRs inhibited the expression of their shared target VEGFA, thereby inhibiting the migration and tube formation of vascular ECs.^47^ Thus, extracting and isolating extracellular vesicles from the wound and detecting the levels of *miR‐195‐5p* or *miR‐205‐5p* in these vesicles may be beneficial for early diagnosis of diabetic wounds.

In the wound of diabetic patients and in human umbilical vein ECs (HUVECs) treated with glucose, the expression of *miR‐133b* is observed to be increased, which leads to the decrease of the expression of its downstream epidermal growth factor receptor, thereby reducing the proliferation, migration, angiogenesis potential of vascular ECs and increasing cell apoptosis.^48^ Hence, *miR‐133b* inhibition may be a useful strategy for treating diabetic wounds.

### 6.2 Altered miRs in EPCs and circulating exosomes

Jie Gao et al^49^ found that in endothelial progenitor cells (EPCs) from diabetic patients, the level of *miR‐155* was increased and the level of PTCH1, its target of action, was decreased, which resulted in decreased cell viability, migration, tube formation, nitric oxide production, lactated hydrogenase, and increased apoptosis. After the protective effect of anti‐*miR‐155* on PTCH1 was applied, ECs function was restored, suggesting that *miR‐155* may be a potential therapeutic target for DFU.^49^

In circulating exosomes naturally occurring in diabetic patients, several studies have found that miRs are abnormally expressed and inhibit EC function. Yuan Xiong et al.^50^ observed that *miR‐20b‐5p* was significantly increased in circulating exosomes from patients with type 2 diabetes mellitus. After acting on the wound, it inhibited the proliferation and tube formation of ECs and enhanced their apoptosis level by inhibiting the Wnt9b/β‐catenin pathway Another group of researchers also found that the expression of *miR‐24‐3p* was also enriched in circulating exosomes from diabetic patients, which targeted PIK3R3 to inhibit the proliferation, migration, tube formation, and autophagy of HUVEC.^35^ Hence, by separating circulating exosomes of diabetic patients and examining the content of *miR‐20b‐5p* and *miR‐24‐3p*, it is helpful for the early diagnosis of diabetic wounds.

### 6.3 MSC‐Exo miRs targeting ECs

Qian Wei et al found that *miR‐17‐5p* was highly abundant in extracellular vesicles secreted by human umbilical cord mesenchymal stem cells. Upon reaching the wound, these vesicles inhibited PTEN expression and activated the AKT/HIF‐1α/VEGF pathway, thereby promoting the proliferation, migration, and tube formation of HUVECs.^51^

Another research found that *miR‐146a* was highly expressed in the extracellular vesicles secreted by mesenchymal stem cells (MSCs), which delayed oxidative stress‐induced HUVECs senescence and increased cell migration and tube formation by inhibiting VE‐cadherin, caveolin‐1 expression, and Src phosphorylation.^52^

Pretreatment of bone marrow mesenchymal stem cells with atorvastatin revealed that exosomes (ATV‐Exos) produced by bone marrow mesenchymal stem cells could act upon vascular ECs, resulting in an upregulation of *miR‐221‐3p*.^53^ After inhibiting the potential target PTEN,^54^ it activates the AKT/eNOS pathway and enhances the proliferation, migration, tube formation, and VEGF expression of ECs.^53^ Therefore, drugs based on the exosome containing the abundant microRNAs mentioned previously are expected to improve the function of local ECs on the wound, and thus improve the repair of the wound.

Table 3 summarizes the specific effects and mechanisms of various miRs mentioned previously on vascular ECs.

**TABLE 3:** The specific effects and mechanisms of various miRs on vascular ECs.

MicroRNA	Source	Change of volume	Target or downstream substances	Change of downstream substances	Proliferation	Apoptosis	Migration	Tube formation	Wound healing time	Supplement
*miR‐152‐3p* ^45^	Wound	↑	PTEN	↓	−	+	/	−	Prolong	
*miR‐199a‐5p* ^46^	Wound	↑	VEGFA, ROCK1	↓	−	/	−	/	Prolong	
*miR‐31‐5p* ^38^	Wound	↓	HIF1AN	↑	−	/	−	−	Prolong	
*miR‐195‐5p* ^47^	Evs from the wound	↑	VEGFA	↓	/	/	−	−	Prolong	
*miR‐205‐5p* ^47^	Evs from the wound	↑	VEGFA	↓	/	/	−	−	Prolong	
*miR‐133b* ^48^	VECs from patients	↑	EGFR	↓	−	+	−	−	Prolong	
*miR‐155* ^49^	EPCs	↑	PTCH1	↓	/	/	−	−	Prolong	Reduces cell viability, nitric oxide production and lactated hydrogenase
*miR‐20b‐5p* ^50^	Circulation	↑	Wnt9b/β‐ catenin	↓	−	+	/	−	Prolong	
*miR‐24‐3p* ^35^	Circulation	↑	PIK3R3	↓	−	/	−	−	Prolong	Enhanced autophagy
*miR‐17‐5p* ^51^	HUMSC‐exosome	↑	PTEN	↓	+	/	+	+	Shorten	Enhanced AKT/HIF‐1α/VEGF pathway
*miR‐146a* ^52^	MSC‐exosome	↑	VE‐cadherin, Caveolin‐1	↓	/	/	+	+	Shorten	By inhibiting Src phosphorylation, delay oxidative stress induced senescence in ECs
*miR‐221‐3p* ^55^	VECs treated by ATV‐Exos	↑	PTEN	↓	+	/	+	+	Shorten	Activated AKT/eNOS pathway and enhanced the expression of VEGF

*Note*: Tips: “↑”: increase, “↓”: decrease, “–”: inhibited, “+”: enhanced, “/”: have no effect or have not been reported in relevant literature.

Abbreviations: EC, endothelial cells; EGFR, epidermal growth factor receptor; EPC, endothelial progenitor cell; HIF‐1, hypoxia‐inducible factor 1; HUMSC, human umbilical cord mesenchymal stem cells; miR, microRNA; PTCH1, protein patched homolog 1; PTEN, phosphatase and tensin homolog; ROCK1, Rho‐associated kinase 1; VEGFA, vascular endothelial growth factor A.

## 7 THE ROLE OF MIRS IN MULTICELLULAR REGULATION AND INTERCELLULAR CROSSTALK

Among the miRs described here, some miRs can be associated with the function of multiple wound cells in the meantime. By intervening these miRs, the function of multiple cells can be restored at the same time. Therefore, these miRs are undoubtedly the best targets for designing drugs that have great potential to improve wound healing.

### 7.1 Multicellular regulation

*MiR‐92a*, targeting the angiogenic integrin α‐5, is present in both human vascular ECs and primary human skin fibroblasts, which can lead to the reduction of angiogenesis and tissue repair ability and delay wound healing.^26^

*MiR‐29b*, the expression of which is low in diabetic wounds, and the level of its negative regulatory target FBN1 is increased. Although this will have a positive effect on fibroblasts,^33^ it will also inhibit the migration ability of keratinocyte (HEKa).^39^ The expression of *miR‐199a‐5p* is increased in diabetic wound tissue, and the levels of VEGFA and ROCK1 regulated by *miR‐199a‐5p* are decreased, thereby inhibiting the proliferation and migration of ECs and fibroblasts.^46^

*MIR‐210*, the low expression of in diabetic wounds, can not only damage the HIF‐1 signaling function in fibroblasts mentioned, but also in human dermal microvascular ECs (HDMECs) and keratinocytes, which may inhibit the proliferation and migration ability of each cell by affecting the glucose metabolism of each cell.^31^ In addition, artificial addition of *miR‐210* can also normalize the prolonged inflammatory phase caused by the upregulation of M1 phenotype of macrophages in wounds, suggesting that the low expression of *miR‐210* may also be related to the high expression of M1 phenotype of macrophages.^31^

*MiR‐146a* was found to be low expressed in both macrophages and keratinocytes, resulting in increased expression of TLR4 and AKAP1, respectively, leading to difficulties in macrophage transformation to M2 phenotype^22^ and inhibition of keratinocyte function.^42^ In addition, bone marrow stem cells‐exosomal vesicles with high expression of *miR‐146a* can improve the function of vascular ECs.^52^ In summary, it can be found that *miR‐146a* plays an important role in a variety of cells in the local wound and shows a positive role in promoting wound repair. Therefore, promoting the expression of *miR‐146* in cells and adding *miR‐146a* mimic are expected to be a very effective intervention to improve wound healing.

### 7.2 Effects on growth factor secretion

#### 7.2.1 VEGF

*MiR‐20b‐5p* is highly expressed in circulating exosomes of diabetic patients, which can not only inhibit the related functions of HSFs but also inhibit the function of vascular ECs through Wnt9b/β‐catenin pathway^50^ and inhibit the secretion of VEGFA by HSFs.^32^ Similarly, overexpressed *miR‐155* in the wound not only inhibits the function of fibroblasts but also further inhibits the expression of VEGF by inhibiting the expression of HIF‐1α in fibroblasts.^19^

Low expression of *miR‐21‐3p* in the wound can increase the expression of its target SPRY1, inhibit the function of fibroblasts and inhibit their secretion of VEGF.^30^ After the reduction of HIF‐1 by *miR‐210*, the secretion of VEGF by keratinocytes was also reduced.^56^ Various miRs reduce the secretion of VEGF through the mechanisms described and then lead to the reduction of related functions of vascular ECs and affect the formation of blood vessels.

#### 7.2.2 Fibroblast growth factor

In addition to VEGF, fibroblast growth factor (FGF) is also a substance that is affected by a variety of miRs and acts on a variety of cells. *MiR‐21‐3p* and *miR‐20b‐5p* can reduce the expression of VEGF and the secretion of basic bFGF in fibroblasts through related mechanisms.^30^, ^32^ Reduced bFGF not only impaired the proliferation of fibroblasts but also impaired the normal function of vascular ECs and keratinocytes.^57^

Overexpression of *miR‐155* in the skin of diabetic mice resulted in inhibition of FGF7 expression.^20^ FGF7 is produced by a variety of cells, such as fibroblasts and ECs, and enhances not only fibroblast proliferation but also keratinocyte proliferation and migration in a paracrine manner.^57^, ^58^ These results suggest that overexpression of *miR‐155* in the skin of diabetic mice can inhibit various cellular functions by inhibiting FGF7 production by fibroblasts and ECs in a paracrine manner. Besides paracrine, *miR‐155* can directly inhibit fibroblasts and inhibit the production of VEGF by fibroblasts, thereby inhibiting vascular ECs. Therefore, inhibiting the overexpression of *miR‐155* in wound is expected to be a means to improve the function of a variety of cells in various ways at the same time, thereby greatly shortening the wound repair time.

### 7.3 Intercellular crosstalk

#### 7.3.1 EC‐Fibroblast

Tingting Zeng et al^59^ found that the expression of *miR‐106b‐5p* was upregulated in extracellular vesicles secreted by ECs after pretreatment with advanced glycation end substances. *miR‐106b‐5p* encapsulated in the extracellular vesicles can act on diabetic wounds by reducing the expression of ERK1/2 in fibroblasts, promoting cell autophagy and reducing collagen synthesis, thereby successfully mediating the inhibitory effect of ECs on the function of fibroblasts in high glucose environment.^59^

#### 7.3.2 EPC‐EC

Juan Xu et al^55^ found that *miR‐221‐3p* is highly expressed in endothelial progenitor cell‐derived exosomes and positively regulates the function of ECs by increasing the expression of VEGF, CD31, and Ki67, thereby mediating the regulatory effect of endothelial progenitor cells on vascular ECs and promoting diabetic wound healing. The finding uncovers the specific mechanism of EPC‐derived exosomes and provides a potential novel approach to the clinical treatment of diabetic skin wounds.

## 8 CONCLUSION

Abnormal expression of a variety of miRs is frequently observed in the wound area or in the blood circulation of diabetic patients. By targeting the relevant molecules of various types of wound cells, numerous cellular functions are often impaired, such as proliferation, migration, autophagy, and so forth. Among them, some miRs are related to the function of multiple cells in the wound. some can act on related targets in multiple cells and affect the function of multiple cells at the same time. Others may only affect one type of cell, but the abnormal function of this cell can lead to abnormalities in multiple other cell types. Certain miRs are also secreted by local wound cells, influencing another cell type and thereby mediating cell crosstalk.

Therefore, it is expected to improve diabetic wound healing by regulating the abnormally expressed miRs, particularly those that can act on multiple cell types, or adding some exogenous miRs and miRs mimics that can enhance cell function. Additionally, gene therapy or cell reprogramming may be conducted on some wound cells to improve the abnormal transcription and secretion of the relevant miRs, thereby directly improving their function or the function of other cells affected by them or the miRs secreted by them, ultimately hastening wound healing.

## DISCLOSURE

All authors declare they have no conflicts of interest.
